# Molecular Mechanisms of DUBs Regulation in Signaling and Disease

**DOI:** 10.3390/ijms22030986

**Published:** 2021-01-20

**Authors:** Ying Li, David Reverter

**Affiliations:** Departament de Bioquimica i Biologia Molecular, Institut de Biotecnologia i de Biomedicina, Universitat Autonòma de Barcelona, 08193 Bellaterra, Spain; liyingbaobei123@gmail.com

**Keywords:** ubiquitin, deubiquitinating enzymes, UPS, proteasome, protein degradation, DUBs, USPs, ubiquitin-code, structural analysis

## Abstract

The large family of deubiquitinating enzymes (DUBs) are involved in the regulation of a plethora of processes carried out inside the cell by protein ubiquitination. Ubiquitination is a basic pathway responsible for the correct protein homeostasis in the cell, which could regulate the fate of proteins through the ubiquitin–proteasome system (UPS). In this review we will focus on recent advances on the molecular mechanisms and specificities found for some types of DUBs enzymes, highlighting illustrative examples in which the regulatory mechanism for DUBs has been understood in depth at the molecular level by structural biology. DUB proteases are responsible for cleavage and regulation of the multiple types of ubiquitin linkages that can be synthesized inside the cell, known as the ubiquitin-code, which are tightly connected to specific substrate functions. We will display some strategies carried out by members of different DUB families to provide specificity on the cleavage of particular ubiquitin linkages. Finally, we will also discuss recent progress made for the development of drug compounds targeting DUB proteases, which are usually correlated to the progress of many pathologies such as cancer and neurodegenerative diseases.

## 1. Introduction

The ubiquitin–proteasome System (UPS) is the major pathway for the degradation of more than 80% of intracellular proteins. Ubiquitination is the major signal to label proteins for degradation, it controls their fates and contributes to the correct cellular homeostasis during all cell cycle phases [1,2]. After a particular protein receives a signal for degradation, such as phosphorylation, DNA damage, protein misfolding, or others, a cascade of ubiquitinating enzymes, namely the ubiquitin-activating enzymes E1, the ubiquitin conjugation enzymes E2, and the ubiquitin ligases E3, labels the protein target with a ubiquitin chain to be carried to the 26S-proteasome, which degrades ubiquitinated-target proteins and recycles ubiquitin for reuse [3]. Several E1, tens of E2 and hundreds of E3 enzymes have been found in human, and they are responsible or the formation of the isopeptide bond between a lysine residue in the target and the C-terminus of ubiquitin. Cells degrade unnecessary proteins in this highly specific manner to prevent cell damage induced by incorrect interaction with other proteins or abnormal protein aggregation. In addition to label proteins for degradation by the UPS pathway, different types of ubiquitin tags can be specifically used by proteins in signaling pathways other than degradation [1,4].

Deubiquitination refers to the process by which ubiquitin molecules are removed from ubiquitin-labeled proteins under the proteolytic action of deubiquitinating enzymes, which cleaves off the isopeptide bond between the C-terminal tail of ubiquitin and the target lysine, thus changing the fate of ubiquitinated proteins and in some instances by preventing them from degradation [5,6]. Deubiquitination has an opposite function to the action of ubiquitin E3 ligases and their balance inside the cell is essential for protein homeostasis and the correct function of the cell. The human genome encodes about 100 deubiquitinase (DUB) genes [7], in contrast to more than 800 genes encoding ubiquitin E3 ligases. These numbers highlight the major role of the ubiquitin pathway in the cell, which must provide a high selectivity grade in the regulation of protein targets.

Ubiquitin can form different types of chain linkages, depending on the lysine residue used in ubiquitin as a target to build poly-ubiquitin chains. Additionally, four different types of protein ubiquitination in substrates enrich the diversity in ubiquitination, namely monoubiquitination, multi-monoubiquitination, homotypic polyubiquitination, and heterotypic polyubiquitination [8,9]. In homotypic polyubiquitination, multiple ubiquitins are linked to each other to form a chain, and are connected to a certain lysine residue (Lys6, Lys11, Lys27, Lys29, Lys33, Lys48, and Lys63) or to the amino-terminal methionine. Also, protein ubiquitination could be accompanied by some other ubiquitin-like modifiers (UBL), such as SUMO, NEDD8, and ISG15. Heterotypic chains include mixed ubiquitin chains and branched ubiquitin chains. In heterotypic polyubiquitination, by generating different connection groups on different ubiquitin molecules, a mixed ubiquitination sequence can be formed. Several modifications can also occur on each ubiquitin, resulting in branched polymers. Due to the diversity of ubiquitinated chain linkage styles, which has been named as “ubiquitin code”, particular DUBs have evolved to have specific preferences for the different types of ubiquitin chains [6,7,10,11].

According to sequence and structure similarity, deubiquitylating enzymes can be divided into seven families, each presenting a unique structural fold: Ubiquitin-specific proteases (USPs), Ubiquitin C-terminal hydrolases (UCHs), Ovarian tumor proteases (OTUs), Machado–Joseph Disease protease family (MJDs), MINDY protease family (MIU-containing DUB family), JAMM family (Jad1/Pad/MPN domain-containing metalloenzymes), and the newly discovered ZUFSP/Mug105 family (zinc finger with UFM1-specific peptidase domain protein/C6orf113/ZUP1) [12,13] (Table 1). Amid them, the JAMM family are zinc-dependent metalloproteinases, while the other six families are cysteine proteases, displaying a characteristic cysteine residue in the context of the active site catalytic triad. Except for MJDs, the other families are highly conserved in yeast and humans (Table 1). Among the 99 DUBs members, 11 are considered pseudo-enzymes due to the lack of key residues for deubiquitinating activity, nevertheless they still can allosterically activate other active deubiquitinating enzymes and other types of active enzymes, thereby executing a critical biological role [14].

UBD (ubiquitin-binding domain) is a type of modular domain that can non-covalently bind to ubiquitin or ubiquitinated substrates, which is widely present in deubiquitinating enzymes. Almost 20 different UBDs families have been discovered so far. According to the structure of UBDs, they are divided into 5 categories: α helix, Zinc finger, PH domain, Ubc-like, and others. The most common in DUBs are UBA (ubiquitin-associated domain), UBL (ubiquitin-like modifiers), UIM (ubiquitin-interacting motif), ZnF-UBP (zinc finger ubiquitin-specific protease domain), and ZnF-A20s (A20-type zinc fingers) [7,15,16]. Recent research has discovered a new UBD named CoCUN [17]. In the process of DUBs recognition and hydrolysis of ubiquitin chains, UBD plays an indispensable role. For example, OTUD1 can specifically hydrolyze the Lys63 ubiquitin chain, but the lack of the UIM domain decreases its specificity [18]. Also, the adjacent UBL domains of USP7 are indispensable to achieve complete deubiquitinating activity [19].

The mechanism by which deubiquitinating enzymes exert their function in the cell can be complex, in some cases it is not only deubiquitination, but it can also promote ubiquitination by recycling ubiquitin molecules, proofreading of the ubiquitination process, and decomposing ubiquitination inhibitors [20]. In this review, we will focus attention on advances in explaining molecular mechanisms and specificities found in some DUBs enzymes, highlighting illustrative examples in which the regulatory mechanism for DUBs has been understood in depth at the molecular level. We will also discuss on DUB specificity and terminate by indicating new advances encouraging their development as therapeutic targets.

## 2. DUBs in Diseases

The large number of deubiquitinating enzymes are not only playing a role in the normal physiological activities of cells, but can also be relevant for the occurrence and development of tumorigenesis and other pathologies [21,22,23]. Target proteins for deubiquitinating enzymes include enzymes, transcription factors, signal transduction molecules, immune response proteins, viral proteins, epigenetic factors, and many other regulators of cell homeostasis, also including products of known oncogenes or tumor suppressor genes [24,25,26,27,28,29,30,31]. Therefore, disease treatment strategies with DUBs as molecular targets have a broad development value and promising clinical application prospects [32]. Many studies have shown DUBs involvement in the regulation of Wnt/β-catenin signaling, TGF-β (transforming growth factor-β), Akt (Protein Kinase B), NF-κB (nuclear factor kappa-light-chain-enhancer of activated B cells) and other cancer-related pathways [33,34,35,36].

Several DUBs are involved in the regulation of the Wnt/β-catenin signaling. For example, overexpression of USP5 will cause an increase in the amount of transcription factor FoxM1, which will increase the content of β-catenin, resulting in faster cell proliferation and carcinogenesis in many tumors [37]. UCH37 can specifically bind and deubiquitinate transcription factor 7 (Tcf7) to activate Wnt signaling in human liver cancer cells [38]. In pediatric high-grade glioma, abnormal expression of UCH-L1 can promote tumor formation [39]. Abnormal activation of USP7 can lead to colorectal cancer [40]. USP14 overexpression is closely related to hepatocellular carcinoma [41]. USP4 is related to colorectal cancer since the overexpression of USP4 exists in many types of tumors, thus it is considered to be a potential oncogene [42,43,44,45]. Most DUBs will promote the occurrence of tumors, but a small number of DUBs play the opposite role. For example, the tumor suppressor cylindromatosis (CYLD), which is expressed in tumor cells, inhibits the proliferation and spread of tumor cells. CYLD is not expressed in multiple myeloma, and inhibits the growth of multiple myeloma by acting on the Dvl substrate [46,47].

Most DUBs reduce the degradation of protein targets by protecting them from the proteasome, thereby a high concentration of TGF-β, promoted by specific DUBs, might lead to tumor occurrence and metastasis. Deubiquitinating enzyme USP10 promotes the metastasis of hepatocellular carcinoma metastasis by deubiquitinating and stabilizing Smad4 [48]. But there are special cases, CYLD can inhibit oral squamous cell in the metastasis of carcinoma, CYLD controls the downstream TGF-β pathway by regulating Smad7, thereby inhibiting the occurrence and development of cancer [49,50].

DUBs also play an important role in regulating the NF-κB pathway. OTULIN/CYLD can interact with LUBAC (linear ubiquitin chain assembly complex) through the PUB (PNGase/UBA or UBX) domain of HOIP (a catalytic subunit of LUBAC), thereby inhibiting the NF-κB pathway and exerting anti-tumor effects [51]. Studies have shown that alterations in the CYLD gene are closely related to HPV-associated cancers, it activates NF-κB and is implicated in invasion and metastasis [52,53,54]. USP15 potentiates NF-κB activation by differentially stabilizing TAB2 and TAB3 [55].

The Akt pathway promotes the survival and proliferation of cells in the organism, and its expression is increased in cancer cells [56]. The increased expression of USP4 will reduce the ubiquitination process of downstream PRL-3 and increase the content of PRL-3, thereby activating the PI3K/Akt pathway. The PI3K/Akt pathway is over-activated, causing tumorigenesis and other diseases [43]. In osteosarcoma cells, overexpression of USP22 leads to increased activity of the PI3K/Akt pathway and causes cancer [57]. USP22 plays a vital role in the development of chemoresistant hepatocellular carcinoma cells [58].

## 3. DUBs Families and Linkage Specificity

### 3.1. Ovarian Tumor-Related Proteases, OTUs

The OTU domain was first discovered in the ovarian tumor gene of *Drosophila melanogaster* by bioinformatics methods [59]. After structural analysis, it was found that although the structure is different from any other known DUB enzyme, it belongs to the cysteine protease class and contained the characteristic catalytic triad. There are 17 OTUs in humans, and members of the OTU family have been revealed to possess obvious recognition specificity for different types of polyubiquitin chain connections, thus disclosing many principles of DUBs chain linkage specificity. For instance, OTULIN cleaves linear ubiquitin [60]. OTUB2 prefers K63 di-ubiquitin substrate [18,61]. OTUB1 only cleaves K48-linked chains [62]. The specificity of OTU family members to recognize polyubiquitin chains of different connection types is likely to be the basis for their recognition of target substrates.

### 3.2. Ubiquitin-Specific Processing Enzymes, USPs/Ubiquitin-Specific Proteases, UBPs

Within the deubiquitinating enzymes, the ubiquitin-specific proteases family (USPs) contains the major number of members (more than 50) and its large structural diversity relies on the presence of multiple domains. All members are cysteine proteases and contain the characteristic catalytic triad in the active site of the catalytic USP domain. The conserved USP domain consists of three subdomains that were initially described as thumb, palm, and fingers of a human right hand in the USP7/HAUSP structure [63]. The catalytic triad of the active site is located between the palm and thumb subdomains, and the finger subdomain is responsible for the interaction with the ubiquitin substrate [64]. In addition to the catalytic domain, other domains of USPs contribute to a large diversity of functions, such as zinc finger domains, ubiquitin-like domains, ubiquitin-associated domain (UBA), ubiquitin-interacting motif (UIM). Some of them are associated with the specific recognition of substrates and poly-ubiquitin chains. Most USP members have no specific linkage-type preference in vitro [65], but in vivo there are some exceptions, for example, CYLD shows K63-linked specificity [66]; USP30 preferentially processes K6-linked chains [67]. Also, some of them show preference for different types of UBL such as USPL1 and USP18 for SUMO and ISG15, respectively [68,69].

### 3.3. Jad1/Pad/MPN Domain-Containing Metalloenzymes, JAMM Family/MPN

The JAMMs is the only family of deubiquitinating enzymes belonging to the metalloprotease family, which contain a Zn atom in the active site. Members of this family are occasionally subunits of large protein complexes, such as COP9 signalosome complex (CSN5 and CSN6 subunits), the 26S-proteasome (PSMD14/Rpn11 and PSMD7/Rpn8 subunits), and the Eukaryotic translation initiation factor 3 (EIF3) (EIF3H and EIF3F subunits) [70,71,72,73,74,75,76]. In this family, AMSH, AMSH-LP, and BRCC36 have been shown to have specificity for K63-linked ubiquitin chains [77,78], and CNS5/COPS5 has activity against NEDD8, another type of ubiquitin-like modifier [79].

### 3.4. Machado–Joseph Disease Proteases, MJDs/Josephin

Currently, four members of the MJDs/Josephin family are found in humans, namely ATXN3/ataxin3, ATXN-3L, JOSD-1 and JOSD-2. All members have a highly conserved catalytic triad formed by one cysteine and two histidine residues. In addition to the Josephin domain, ATXN3 and ATXN3L also have ubiquitin interaction motifs (UIMs), which are likely to mediate the interaction between protein and ubiquitin in protein multimers [80]. ATXN3 can bind both K48 and K63-linked ubiquitin chains, but preferentially shows activity for K63 linkages [81]. ATXN3L binds to K48 and K63-linked ubiquitin chains in vitro [82]. JOSD1 only has activity for monoubiquitin and JOSD2 cleaves K48 and K63-linked ubiquitin chains in vitro [83].

### 3.5. Ubiquitin C-Terminal Hydrolases, UCHs

UCHs are also cysteine proteases, including UCHL1, UCHL3, UCHL5/UCH37, and BAP1 (BRCA 1 associated protein-1). Substrates are usually small proteins like short polypeptides or small protein domains. The narrow pocket on the active site of UCHs and the limitation of the diameter of the loop precludes binding and catalysis of large ubiquitinated proteins, to a certain extent [84,85,86]. UCH-L1 can cleave short peptides from the C-terminus of ubiquitin in vitro [87]. UCH-L3 cleaves the C-terminus of NEDD8 or ubiquitin [88]. UCH-L5 participates in the formation of 19S regulatory particles, it can hydrolyze ubiquitin chains [89]. BAP1 can also regulate the polyubiquitination of the transcriptional cofactor HCF-1 (host cell factor 1) [90].

### 3.6. MIU-Containing Novel DUB Family, MINDY

MINDY family members, include MINDY1, -2, -3, -4, and -4B in humans, are specific for K48-linked ubiquitin chains [91]. The crystal structure of MINDY1 exhibited a novel protein fold without homology to any other type of DUB [91]. MINDY-1 prefers to cleave long polyubiquitin chains and cleaves the distal ubiquitin component [7,91,92]. In the absence of the substrate, the active site in MINDY-1 has a non-productive conformation, which is remodeled and the catalytic triad rearranged upon substrate binding [91].

### 3.7. Zinc Finger with UFM1-Specific Peptidase Domain Protein/C6orf113/ZUP1

ZUP1/ZUFSP in humans and Mug105 in fission yeast are representative members of a seventh DUB family. The ZUFSP family seems equidistant to the UFSP and ATG4 families, and the catalytic domain is related to the UFM1-protease UFSP2, but ZUFSP is not a member of UFSP [93]. Whereas the UFM1-specific protease UFSP2 has no detectable DUB activity, ZUFSP has linkage-selective DUB activity [12]. Human ZUFSP selectively interacts and cleaves long K63-linked ubiquitin chains using tandem ubiquitin-binding domains, but it displays poor activity toward mono- or di-Ub substrates. Mug105 prefers K48-linked chains [12,94]. The discovery of other members of the family will probably assist the analysis of the linkage specificity, as well as to shed light on structural and functional properties.

## 4. Regulatory Mechanisms of DUB Activity and Specificity

Research on deubiquitinating enzymes shows that although deubiquitinating enzymes are classified into several distinct families, the structural differences between members have caused their functional diversity. Here, we have selected a few classical, special, or interesting examples published in recent years to explain the molecular mechanism of DUB regulation at the molecular level, based on their crystal structures. These in-depth examples of DUB regulation may inspire researchers to shed light on deubiquitinating enzymes with still unknown mechanisms.

### 4.1. USP7, a Conformational Rearrangement of the Catalytic Triad

**Figure 1:**
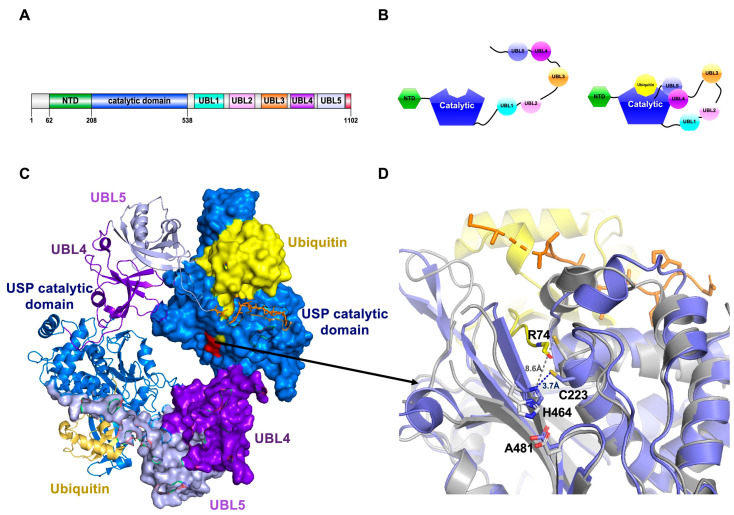
Active site rearrangements in USP7. (**A**) Schematic representation of the USP7 domain organization. (**B**) Model for the activation of USP7 by ubiquitin and ubiquitin-like modifiers (UBL) domains (different colors). (**C**) Crystal structure of the dimer of the USP7CD-UBL45-ubiquitin complex. Ubiquitin (yellow), the C-terminal peptide after UBL5 (orange). (**D**) Comparison of the active sites between USP7CD-UBL45-Ub and USP7CD. Active USP catalytic domain (blue), non-active USP catalytic domain (gray). Dotted lines are distances between active site Cys and His residues (PDB: 5JTV, 5FWI).

USP7/HAUSP has been deeply studied from the structure/function standpoint and its molecular signaling pathway is well established and considered a potential therapeutic target for cancer [95]. The human USP7 gene is located on chromosome 16, contains 1102 amino acid residues and is mainly located in the nucleus, but also slightly distributed in the cytoplasm [95]. USP7 is highly conserved in mammals, with a 98.6% sequence identity between human, mouse and rat [96]. USP7 is a multidomain protein with seven domains: In addition to the “canonical” USP catalytic domain (aa208-560), it contains one N-terminal TRAF-like domain (Tumor necrosis factor Receptor-Associated Factor), and five consecutive C-terminal UBL domains (Ubiquitin-like domains) [97] (Figure 1A,B). UBL domains can be also found in other USPs and are known to play a role in the regulation of the enzymatic activity [98]. USP7 can cleave K6, K11, K33, K48, and K63-linked ubiquitin chains [63,99].

The initial crystal structure of the catalytic domain of USP7 defined the characteristic right hand-like structural fold of the USP family, containing the active site catalytic triad: Cys223, His464, and Asp481. Remarkably, in contrast to other USPs, the cysteine and histidine of the USP7 catalytic domain are in a non-productive configuration in the apoenzyme and need to be activated by structural rearrangement upon ubiquitin-binding [63,98] (Figure 1B,C). The N-terminal TRAF-like domain and the C-terminal UBL domains are important sites for the binding of USP7 to other proteins [63]. USP7 can interact with p53, HDM2, HDMX, MCM-BP, UbE2E1, vIRF1, vIRF4, and EBNA1 through the TRAF-like domain [100,101,102,103,104,105,106].

Interestingly, the active site of USP7 shows an inactive conformation in its apo form, but in the presence of ubiquitin, the active site is remodeled and the catalytic triad aligned [64] (Figure 1D). UBL domains also play a role in the activation of the catalytic domain [107]. Deletion of the USP7 C-terminal UBL domains have an impact on the USP7 activity and then USP7 cannot bind ubiquitin chains [19,97]. According to the structure of the USP7CD-UBL4-5-Ubiquitin complex (Figure 1C), the C-terminal extension after UBL-5 domain binds a cavity next to the active site and promotes its activation. The C-terminal peptide seems to stabilize the active conformation instead of significantly change the structure of the catalytic domain. It is accommodated in a hydrophobic cavity by several contacts, such as Ile1098 and Ile1100, and can also bind the C-terminal Arg74 of ubiquitin [97]. This regulatory C-terminus extension of USP7 is essential for the enhancement of the USP7 catalytic activity [97,108].

Among all C-terminal UBL domains, the presence of the UBL4-5 domain is enough to reconstitute the USP7 activation in vitro [19]. USP7 activation can be triggered by interaction with other substrates to the C-terminal UBL domains, such as binding to GMPS (guanosine 5′-monophosphate synthetase) [19]. Other USP7 substrates also bind to the UBL domains, such as DNMT1, ICPO, RNF169, and UHRF1 [109,110,111,112,113]. Therefore, the C-terminal UBL domains have a role in the regulation of USP7 by inhibiting or enhancing enzyme activity [104]. In the cell, different substrates compete to bind to the same surface on USP7 [100].

USP7 has been related to many diseases, such as lung cancer, cervical cancer, herpes simplex virus, and nasopharyngeal carcinoma by Epstein–Bar virus (EBV) [114,115,116,117]. USP7 plays an important role in tumorigenesis and progression by deubiquitinating a variety of tumor regulatory molecules [95] and is a promising target for the development of lead compounds.

### 4.2. Ubp8, Needs Partner Proteins for Activity

**Figure 2:**
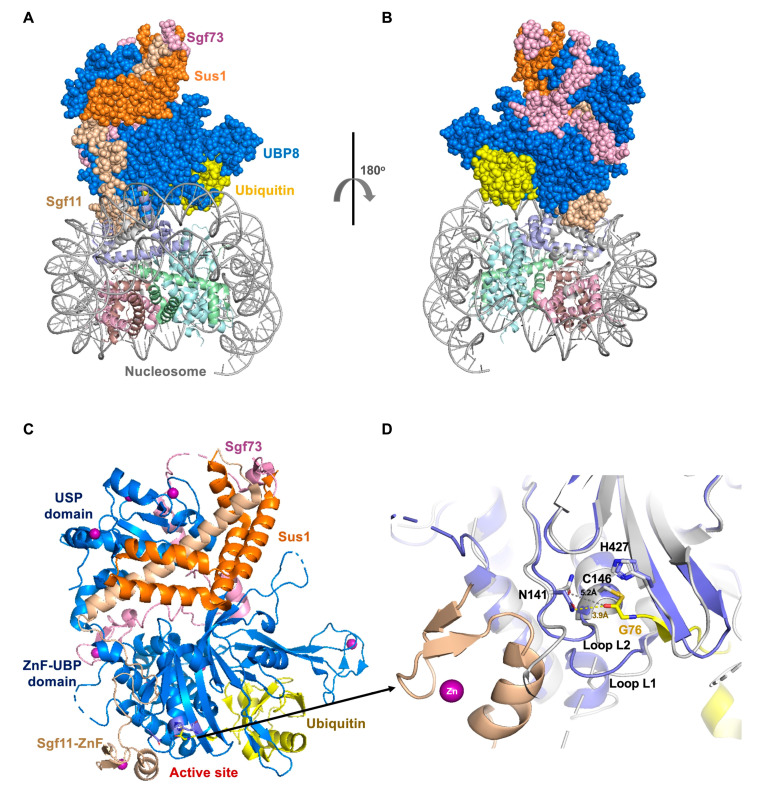
The structure of Ubp8 in the SAGA deubiquitinating module. (**A**) Crystal structure of the SAGA DUB module in complex with monoubiquitinated nucleosome (PDB: 4ZUX). (**B**) View of the complex rotated 180°. (**C**) Structure of Ubp8/Sgf11/Sus1/Sgf73 bound to ubiquitin aldehyde (PDB: 3MHS). (**D**) Zoom-up and superposition of the active site with or without the Sgf11-ZnF domain. Ubp8 (blue), Sus1 (orange), Sgf73 (pink), sgf11 (wheat), ubiquitin (yellow), zinc atom (purple) (PDB: 4FJC and 3MHS).

Ubp8, the yeast protein homolog of human USP22, is a subunit of the SAGA complex, which is a multifunctional complex protein involved in many biological processes such as transcription activation and mRNA transport [118]. The center module for catalyzing deubiquitination is called the DUB region and contains four subunits: Ubp8, Sgf11, Sus1, and Sgf73 [119,120,121] (Figure 2A,B). Ubp8 deubiquitinates histone H2B [122]. Ubp8 can sufficiently perform its deubiquitinating activity with the assistance of the other partners of the complex: Sgf11, Sus1, and Sgf73 [123].

The active site residues of Ubp8 are catalytically well-arranged in the absence of ubiquitin [119,121,124]. However, the ZnF-UBP (zinc finger-ubiquitin binding) domain of Ubp8 lacks vital residues for ubiquitin-binding, since the ubiquitin-binding area is at the interface with the Sgf11 and Sus1 subunits, it needs other proteins of the complex to help its interaction with ubiquitin [98,124,125] (Figure 2C). SAGA promotes deubiquitination through the specific binding of the zinc finger domain of Sgf11 to the acidic pocket of ubiquitinated nucleosomes [124]. Moreover, Sgf11 plays a regulatory role in stabilizing the active conformation of the catalytic active site of Ubp8 [121]. The C-terminal zinc finger domain of Sgf11 (Sgf11-ZnF) binds to the Ubp8 USP domain next to the catalytic site, making extensive contacts with the loop containing the active site residues (called loop L2) (Figure 2D). The N-terminal 100 residues of Sgf73 connects the DUB domain with other parts of the SAGA complex and regulates the spatial structure of the complex [121,126]. The zinc fingers of Sgf11 and Sgf73 are essential for the activity of the DUB module: Deleting Sgf11-ZnF will greatly reduce the activity of the enzyme, while the absence of the zinc finger of Sgf73 will cause Sgf73 to dissociate from the DUB module, thereby abolishing the deubiquitinase activity [124,127]. It has been proposed a role for Sgf73, Sgf11, and Sus1 in stabilizing a conformation of Ubp8 that can easily interact with ubiquitin [119].

Overexpression USP22 is a marker of aggressive cancer phenotypes like metastasis, and therapy resistance studies have shown that catalyzing SAGA subunit USP22 to control CCND1 ubiquitination is important for the progression of cancer cells through the G1 cell cycle [128]. USP22 promotes the advance of gastric cancer by stabilizing BMI1 protein [129]. Also, USP22 plays a carcinogenic effect in prostate cancer by regulating cell proliferation and DNA repair [130].

### 4.3. USP25, an Auto-Inhibitory Homotetramer

**Figure 3:**
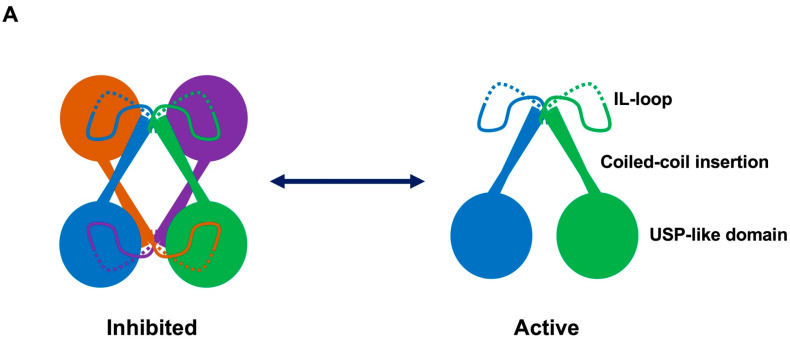

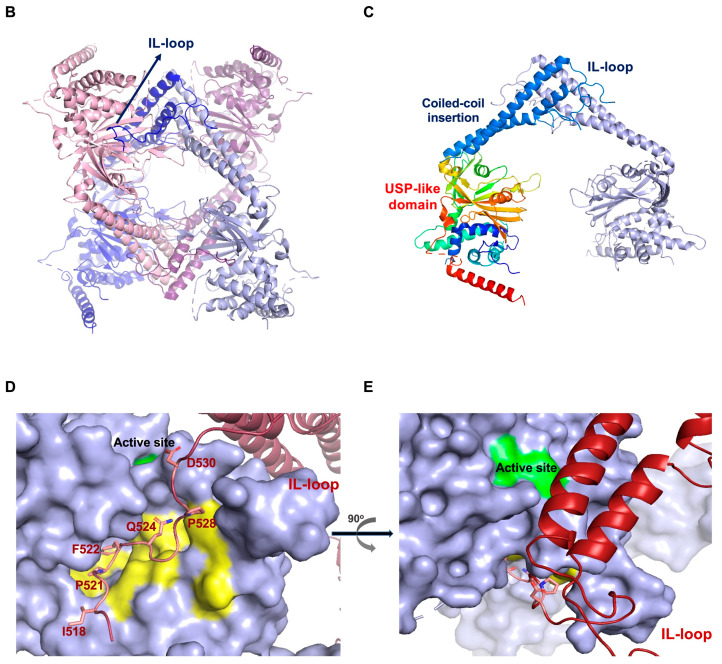
The structure of USP25. (**A**) Schematic cartoon representation of the USP25 tetramer and dimer assemblies. Each monomer is colored. (**B**) Crystal structure of the USP25 tetramer (PDB: 5O71 and 6HEL). Each dimer is colored blue or light pink. (**C**) Crystal structure of the USP25 dimer. IL-loop, coiled-coil insertion and ubiquitin-specific protease (USP)-like domain are marked. (**D**) Binding interface of the IL-loop with the ubiquitin-binding site of USP25. IL-loop (red), active site (green), binding surface (yellow), the interface contacts of IL-loop are labelled. (**E**) View of D rotated 90°.

USP25 is a special member of the USP family due to its auto-inhibitory mechanism. The 180 amino acids insertion in the middle of the catalytic domain of USP25 plays a crucial role in the catalytic activity. There are two conformations of USP25: Tetramer and dimer. The dimer USP25 is active, while the tetramer USP25 has a low activity [131] (Figure 3A–C). According to structural biology analysis [131,132,133], an inhibitory loop binds the ubiquitin-binding surface of the USP domain in the tetramer conformation, resulting in a failure of USP25 to cleave ubiquitin substrates. USP25 can switch in the cell between dimer (active) and tetramer (inactive) [133]. This special feature is unique to USP25, and it has not been found in any other USP member to date.

USP28 has 57% amino acid sequence identity with USP25, but USP28 is not regulated by this autoinhibitory mechanism. USP28 has always a dimeric conformation, never a tetramer [133]. The unique coiled-coil helical insertion domain in the middle of the catalytic domains of USP25 and USP28 is essential for the formation of dimers [132] (Figure 3D,E). Probably due to the slightly different orientation of the dimer, USP28 cannot form a tetramer structure. Therefore, despite USP25 and USP28 possess high sequence and structural similarities, they display different regulatory mechanisms.

Other USP members, such as USP4, USP7, and USP14, also have a certain degree of auto-inhibition, but not through tetrameric inhibitory conformations like USP25. Studies have shown that the activity of USP14 is significantly improved after binding the proteasome [134,135]. The DUSP-UBL domain at the N-terminus of USP4 plays an indispensable role in the catalytic activity of USP4, since the dissociation rate of ubiquitin will affect the catalytic activity of USP4, the DUSP domain can promote the dissociation of ubiquitin, thereby improving the catalytic activity of USP4 [136].

USP25 might be an oncogene, it participates in the stabilization of tankyrases that can regulate oncogenic pathways and have an impact on the invasion and migration of cancer cells [137,138]. A latest research shows that USP25 is a new target to induce degradation of oncogenic BCR-ABL protein in Ph-positive leukemia cells [139].

### 4.4. OTUB1, a Non-Canonical Inhibition of the E2-Conjugating Enzyme

OTUB1 (OTU domain-containing ubiquitin aldehyde binding protein 1) is an extensively studied member of the OTU deubiquitinating family. The human OTUB1 gene is located in chromosome 11 and encodes an enzyme of 271 residues [140]. Its subcellular location is regulated by casein kinase 2 (CK2), which phosphorylates Ser16 of OTUB1 moving it from the cytoplasm to the nucleus [141]. OTUB1 can participate in the regulation of p53 stability, immune response, signal transduction, DNA damage repair, and other biological processes [142,143,144,145,146]. OTUB1 is a K48 specific deubiquitinating enzyme, and in addition to the DUB activity, OTUB1 has also emerged as a special DUB that can suppress the activity of E2 conjugating enzymes, inhibiting thus the ubiquitination process [143].

**Figure 4:**
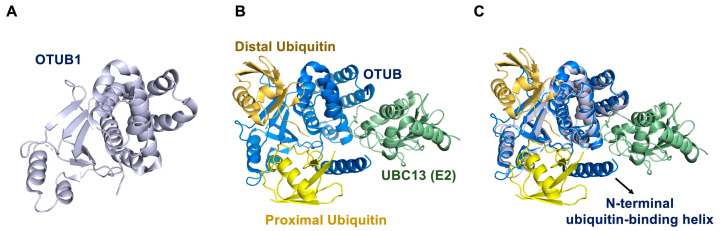
The structure of OTUB1 and E2. (**A**) Crystal structure of OTUB1. (**B**) Crystal structure of OTUB1-ubiquitin aldehyde-UBC13~Ub. (**C**) Superposition of OTUB1 structures in (**A**,**B**). Ubiquitin (yellow), UBC13 (green), OTUB1 without ubiquitin (white-blue), OTUB1 with ubiquitin (blue), N-terminal ubiquitin-binding helix (blue) is disordered in the apoenzyme. (PDB: 4DHZ and 2ZFY).

Researches have convincingly illustrated a unique mechanism for OTUB1 performing different biological functions through the interaction with E2-conjugating enzymes (such as UBC13 and UBCH5) [147,148]. OTUB1 contains two different specific binding sites for ubiquitin: The proximal ubiquitin-binding site binds the donor ubiquitin, and the distal ubiquitin-binding site binds free ubiquitin [149,150] (Figure 4A,B). E2 binding to OTUB1 stabilizes an N-terminal disordered helix that interacts with the proximal ubiquitin and enhances the deubiquitinating activity of OTUB1 for K48-linked chains (Figure 4A–C). The affinity of OTUB1 for the charged E2-Ub is increased when a free ubiquitin monomer binds to the distal site of OTUB1 [147,148,149]. However, when E2 is charged with the ubiquitin thioester linkage, it locates its donor ubiquitin in the proximal ubiquitin-binding site of OTUB1, and in this scenario, the deubiquitinating activity of OTUB1 for K48-linked chains is inhibited and the charged E2 cannot deliver ubiquitin to substrate and the ubiquitination conjugation activity of E2 is inhibited. Also, free ubiquitin binding to the distal site of OTUB1 has been shown to compete with the binding of K48-linked chains and inhibits the deubiquitinating activity of OTUB1 [148]. Likewise, binding of uncharged E2s to OTUB1 stimulates OTUB1 cleavage of K48-linked substrates [150]. Thereby, the relative proportion of E2 versus E2~Ub and the local concentration of K48-linked polyubiquitin chains as well as free ubiquitin decide if OTUB1 plays a role either as DUB or as an inhibitor of ubiquitination [150].

Consequently, in addition to its DUB activity, OTUB1 also has the special function to bind different E2 ubiquitin conjugation enzymes, causing inhibition of ubiquitin transfer and regulation ubiquitination in a non-catalytically manner. Recently, another role for OTUB1 has been found by the binding of the E2-conjugating enzyme UBE2E1 (UBCH6) [151]. UBE2E1 is ubiquitinated in vivo and can be targeted to the proteasome for degradation. In vitro, OTUB1 can non-catalytically inhibit the autoubiquitination activity of UBE2E1, thus preventing it from degradation [149]. Probably, more mechanisms for the regulation of other E2 conjugation activities by OTUB1 will be disclosed in the future.

OTUB1 is involved in many cancers [142]. For example, OTUB1 regulates epithelial-mesenchymal cell transformation through the PI3K-AKT-GSK3β signaling pathway in colorectal cancer [141]. OTUB1 activates RhoA to promote prostate cancer cell invasion and promote tumorigenesis in prostate cancer [152]. OTUB1 represses the ubiquitination and degradation of FOXM1 in breast cancer [146]. The isoform OTUB1 promotes G1-S phase transition, inhibits cell apoptosis, and promotes tumor cell proliferation as well as invasion in gastric cancer [153]. But so far, there are no specific inhibitors for OTUB1.

### 4.5. OTULIN, with a Little Help of Ubiquitin for Activation

OTULIN (OTU domain DUB with Linear Linkage specificity), also known as Gumby/FAM105b, is a member of the ovarian tumor-associated protease family (OTUs) and it is a deubiquitinating enzyme that specifically hydrolyzes Met1-Ub linear linkages [154]. The human OTULIN gene is located in chromosome 5 and encodes a protein of 352 amino acids [18,36]. OTULIN can actively cleave the connection point between Met1 and Ub in the linear chain, but it has a very weak activity in the connection point between ubiquitin and the substrate lysine.

**Figure 5:**
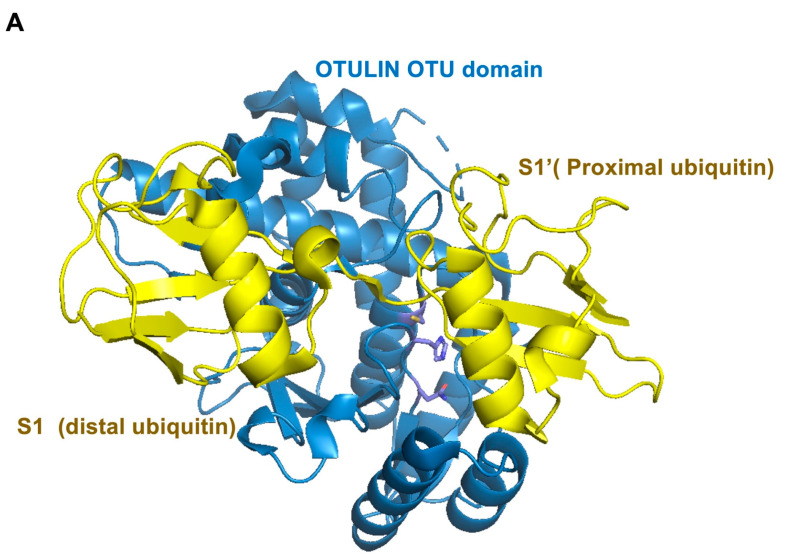

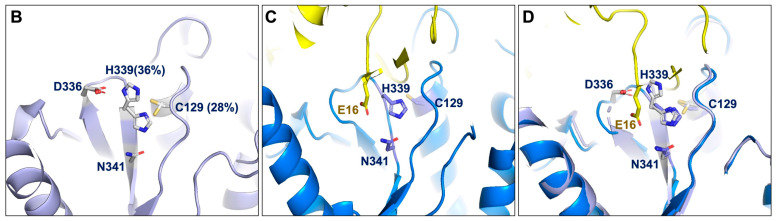
The structure of OTULIN. (**A**) Structure of OTULIN OTU domain in complex with Met1-di-ubiquitin. OTULIN (blue), ubiquitin (yellow). (**B**) Rearrangement of the active site residues in the absence (white blue) of bound Met1-di-ubiquitin. (**C**) Rearrangement of active site residues in the presence (blue) of bound Met1-di-ubiquitin. (**D**) Superposition of (**B**,**C**). (PDB: 3ZNZ and 3ZNV).

The distal and proximal Met1-Ub linear linkages can respectively interact with the ubiquitin-binding sites S1 and S1’ (Figure 5A). Point mutations in S1 or S1’ will reduce the affinity of OTULIN for Met1-Ub, thereby reducing the DUB activity of OTULIN [154]. In the absence of substrate, the histidine and cysteine residues of the catalytic triad of OTULIN (Cys129, His339, and Asn341) display two alternating conformations [155], with occupancy rates of 28% and 36% for cysteine and histidine, respectively (Figure 5B), exhibiting an auto-inhibited active site conformation.

Interestingly, the interaction of the linear ubiquitin linkage substrate to the active site of OTULIN is essential for activation. Indeed, the proximal ubiquitin directly participates in the activity of the catalytic center. Particularly, the carbonyl group of Met1 interferes with the non-productive conformation of His339, thereby changing it to an active conformation (Figure 5C,D). Ubiquitin Glu16, at the binding site of Met1-Ub and OTULIN, has a decisive effect on catalytic activity. The Glu16 side chain of the proximal ubiquitin is inserted into the active site, replacing the position of Asp336, and further restricting the His339 conformation [154]. Glu16 and Asp336 prompts His339 into an active conformation to further deprotonates Cys129 [156]. At the same time, Glu16 cooperates with Asn341 to further increase the catalytic activity of the catalytic center. Mutation of Glu16 to Ala reduces the catalytic efficiency of OTULIN by 240-fold [154,155].

OTULIN regulates immune receptor signaling by interacting with LUBAC. OTULIN deficiency results in severe auto-inflammatory disease in patients [157]. OTULIN can protect the liver against cell death and cancer, OTULIN deficiency in non-hematopoietic cells causing liver pathology [156,158]. OTULIN inhibits liver inflammation and hepatocellular carcinoma via restraining FADD- and RIPK1 kinase-mediated hepatocyte apoptosis [159]. Recent studies have shown that OTULIN may related to HIV-1 activation [160].

### 4.6. RPN11, Cleaves the Ubiquitin Chain in the 26S Proteasome

There are three important deubiquitinating enzymes in human proteasomes: USP14, UCH37, and Rpn11 [2]. Rpn11 is a Zn^2+^ dependent protein of the JAMM/MPN protein family [161], which is located straight over the gate channel of the 20S proteasome center [162]. The 26S proteasome mainly recognizes ubiquitinated target proteins through the two intrinsic ubiquitin receptors Rpn10 and Rpn13, and cleaves the 48-linked polyubiquitin chain by the deubiquitinating activity of Rpn11, which assists the 20S proteasome core particle to degrade proteins [163,164]. Rpn11 is activated by proteasome association as well as by ATP hydrolysis [165].

**Figure 6:**
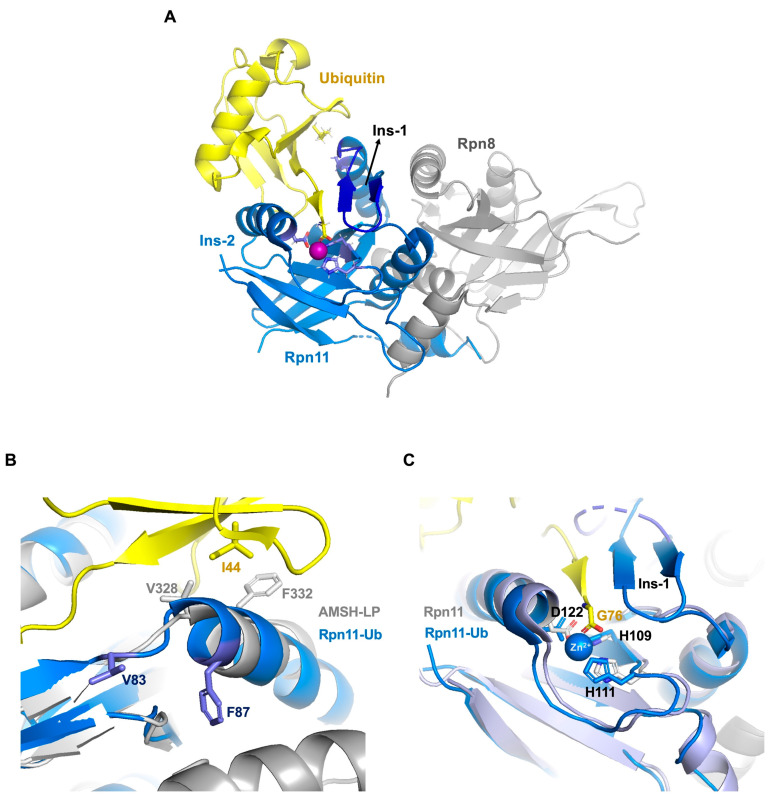
Structure of Ubiquitin-Bound Rpn11-Rpn8. (**A**) Structure of Rpn11-Rpn8 with ubiquitin. Ubiquitin (yellow), Rpn11 (blue), Rpn8 (gray), Zinc (purple) (**B**) Comparison of conserved binding sites of Rpn11 and AMSH-LP for the Ile44-ubiquitin-binding surface. Rpn11 (blue), AMSH-LP (gray). Val83 and Phe87 are from Rpn11, Val328 and Phe332 are from AMSH-LP. (**C**) Comparison of the active sites of Rpn11 and Rpn11-Ub. (PDB: 5U4P, 5W83, 4O8X and 2ZNV).

Rpn11 is hard to purify alone, but in complex with Rpn8 forms a stable and functionally DUB heterodimer [166,167] (Figure 6A). Rpn8 and Rpn11 are canonical MPN domains, but Rpn8 is catalytically inactive and does not contain the JAMM Zn-binding motif and it has mainly a supporting role for Rpn11 [166]. Also, Rpn11 does not show complete features of a fully active DUB structure, it lacks conserved residues responsible for ubiquitin-binding and stabilization of the isopeptide bond for cleavage [166,167].

Rpn11 and AMSH-LP bind ubiquitin in a similar orientation, but their interaction with the Ile44 hydrophobic patch is quite different. It is known that Val328 and Phe332 of AMSH-LP interact with ubiquitin Ile44, but structural comparison shows that the equivalent positions in Rpn11 (Val83 and Phe87) are oriented in an opposite direction from AMSH-LP (Figure 6B). Phe87 of Rpn11 does not bind ubiquitin but participates in stabilizing the heterodimer interface of Rpn11 and Rpn8, which may help to place Rpn11 above the central pore of the proteasome and allow translocation-coupled substrate deubiquitination [167].

Ubiquitin binding to Rpn11 does not require substrate interaction with the proteasome RP (regulatory particle) [168]. The MPN domain of Rpn11 has three essential regulatory regions: Insertion 1 (Ins-1), Insertion 2 (Ins-2) and the catalytic loop. Ins-1 and Ins-2 are necessary for activity in many MPN domain [77]. Ins-2 region determines the DUB specificity for a specific ubiquitin connection type by interaction with the proximal ubiquitin [77]. However, *Ins-2* of Rpn11 is not ordered and does not interact with the proximal ubiquitin (Figure 6A) [167].

In other JAMM members, the Ins-1 loop has a permanently active β-hairpin structure, such as in AMSH or AMSH-LP, but the Ins-1 loop of Rpn11 is closed and inactive. According to the crystal structure of the Rpn11-Rpn8 heterodimer bound to ubiquitin, the Ins-1 loop of Rpn11 changes its conformation with different states of the proteasome, before and after ubiquitin-binding, from the closed state to the β-hairpin active state [169]. The Ins-1 loop is required for DUB activity [76,169]. The newly formed β-hairpin and residues Arg74 and Gly75 from ubiquitin constitute an anti-parallel triple-strand β structure, thereby placing the scissile isopeptide bond at the active site for cleavage [169] (Figure 6A,C).

Rpn11 is an important potential target for drug therapy [161]. RPN11 may function as an oncogene that promotes proliferation and migration of breast cancer cells [170].

### 4.7. USP12-UAF1-WDR20, Help of Partners for Activation

**Figure 7:**
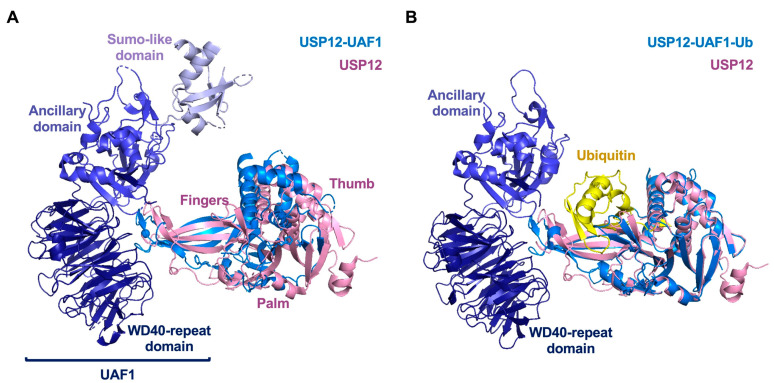

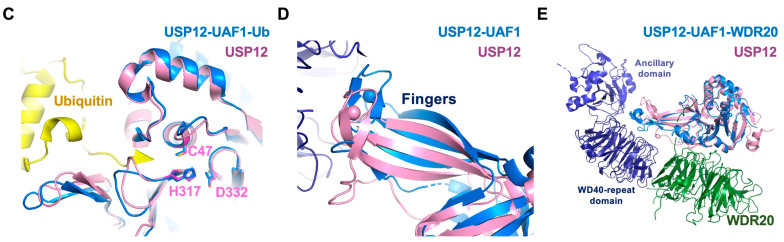
Structure of USP12-UAF1-WDR20. (**A**) Structure of with USP12-UAF1. (**B**) Structure of with USP12-UAF1(9-580)-Ub. (**C**) Active site of USP12. (**D**) Differences of Fingers domain in the absence and presence of UAF1. (**E**) Structure of USP12-UAF1(9-580)-WDR20. Free USP12 (pink), UAF1(deep blue), WDR20 (green), ubiquitin (yellow). (PDB: 5K16, 518W, 5K1B, and 5K1C).

Prostate cancer (PC) is a common male malignant tumor [171]. Androgen receptor (AR) plays a key role in regulating the gene transcription program of PC [172]. USP12 is a co-activator of androgen receptor involved in PC through deubiquitination and stabilization of AR [24,173]. USP12 (370 amino acids) shows a high degree of homology (88%) with Usp46 (366 amino acids). USP1-associated factor 1 (UAF1) is a 80KDa multidomain protein formed by an N-terminal WD40 repeat domain, a Central Auxiliary domain and a C-terminal SUMO-like domain [174,175] (Figure 7A). UAF1 is a USP modulator that can bind to and activate three human deubiquitinases: USP1, USP12, and USP46 [176,177]. The active site of free USP12 has a catalytically active conformation, but its activity is low. The narrow catalytic cleft of free USP12 needs to undergo a conformational change to accommodate ubiquitin [174].

UAF1 interacts with the distal end of the USP12 Finger domain, causing a series of structural changes in USP12, which are then transmitted to the essential ubiquitin contact loop adjacent to the catalytic cleft, thereby enhancing the activity of USP12 (Figure 7B,C) [174,177]. The interaction of UAF1 can stabilize the outer edge of the Fingers domain, transforming it from an unstable state into a regular β chain, completing the four-stranded β structure of the Fingers domain [174]. Mutation analysis confirmed the important role of the Fingers domain in USP12 for its activation by UAF1 binding [178] (Figure 7D).

WDR20 (WD repeat-containing protein 20) is a single domain protein containing WD40-repeat motifs [174]. USP12 can be further activated by WDR20 [179,180]. WDR20 interacts with the USP12 Palm domain, and it is paralleled with the WD40 repeat domain of UAF1 (Figure 7E). The binding sites of UAF1 and WDR20 are located on an opposite surface to the catalytic triad of USP12, but they can allosterically activate USP12 through different structural rearrangements. UAF1 activation of USP12 requires structural fine-tuning at the ubiquitin-binding site, while WDR20 directly regulates the catalytic center of the enzyme remotely, transforming USP12 into a compact structure similar to USP46-Ub [174].

Interestingly, it has been shown that USP12-Ub and UAF1 have two possible binding sites: The Finger domain and the back of the ubiquitin-binding cleft [178]. The second UAF1 binding site has low affinity and partially overlaps the WDR20-USP12 binding interface, thus the binding of WDR20 to USP12/UAF1 may prevent the second UAF1 molecule from binding to USP12, so it may have a regulatory effect [178].

Also, UAF1 can interact with USP1 and USP46 in addition to USP12 [177]. USP1 is considered an important drug target [181]. USP1 was the first deubiquitinating enzyme found to enhance its activity through UAF1 interaction, displaying an increase up to 35-fold [176]. The USP1-UAF1 protein complex is an important regulator in DNA damage response pathways [182].

In addition to PC, USP12 is involved in other diseases. USP12 is a potential target for the treatment of Hepatocellular Carcinoma (HCC), which can prevent G2/M and trigger apoptosis [183]. Also, USP12 causes autophagy and confer neuroprotection in Huntington’s disease (HD) [184].

## 5. Conclusions

In this review, we have introduced and described the mechanisms for the catalytic regulation in different types of DUBs. Most of the discovered DUBs have a similar basic mechanism of deubiquitinating activity for cleavage of the isopeptide bond, but with the presence of regulatory domains adjacent to the catalytic domain or by the formation of tetrameric complex assemblies, DUBs have developed specific strategies to regulate the interaction and cleavage of the different types of linkages of the ubiquitin-code, which will probably reveal layers of complexity that are still not completely understood.

Deubiquitinating enzymes play an essential role in the process of life, and the occurrence and development of many diseases are directly related to them [21,22,23]. The in-depth study of the molecular mechanism of deubiquitination will help us to understand and treat the physiological functions and diseases. Therefore, research on deubiquitinating enzymes has always attracted much attention to the pharmaceutical industry. Research in recent decades has shown that deubiquitinating enzymes can regulate multiple signaling pathways [33,34,35,36], suggesting to researchers the development of new medicines based on the pathogenesis of different diseases that can target deubiquitinating enzymes. For example, the development of deubiquitinating enzyme inhibitors [185], especially selective inhibitors, may have a good therapeutic effect [186,187].

As one of the most well-studied DUBs, USP7 has many small molecule inhibitors. USP7 is currently an important targeted protein for tumor therapy. Studies have found that USP7 inhibitors P5091 and Parthenolide inhibits Wnt signaling and colorectal tumor growth; specific inhibitor HBX 19818 can selectively reduce the activity of USP7; USP7 small-molecule inhibitors P5091 and P22077 can induce tumor cell apoptosis by enhancing intracellular oxidative stress response and endoplasmic reticulum stress response [40,188,189,190,191]. Structural biology helped to reveal some inhibitory mechanisms of small molecule inhibitors on DUBs. As ubiquitin-competitive small molecules, FT671 and FT827 have high affinity and specificity for inhibiting USP7, they target the dynamic pockets nearby the catalytic center of the apo-USP7 [192]. FT671 extends into the Fingers subdomain and catalytic center of USP7, reducing the stability of the USP7 substrate, leading to P53 target gene transcription and tumor growth inhibition in mice. FT827 extends to the catalytic center and forms a covalent bond with the catalytic Cys223 and thus inhibiting the activity of USP7 [192]. XL188 is another selective inhibitor of USP7, which occupies some subsites in the substrate-binding cleft, resulting in the increase of tumor suppressor proteins P53 and P21 [193]. Other DUBs-related inhibitors have also been discovered, for example, capzimin is a potent and specific inhibitor of proteasome isopeptidase Rpn11, which can stabilize proteasome substrates and block the proliferation of cancer cells [194]. A specific inhibitor of USP14, b-AP15, can significantly increase the apoptosis of leukemia cells [195]. IU1-47 is a specific inhibitor of USP14, which inhibits tumors by reducing the expression of tau protein in cells [196]. There are also some newly discovered USP14 candidate inhibitors [197,198]. ML323 is a highly effective inhibitor of USP1-UAF1 deubiquitinase complex, blocking the allosteric activation pathway may be another strategy to study and discover USP inhibitors [178,199,200].

It is worth noting that there are still many unsolved problems in the research of deubiquitinating enzymes, and it is likely that novel regulatory mechanisms, similar to the examples depicted in this review, will be uncovered for other DUBs in the future. The development of inhibitory DUB compounds, to treat tumor growth and other pathologies, are based on a better understanding of the structure and regulatory mechanism in the context of each particular DUB. Research on DUB inhibitor drugs has made some progress, but more efforts will be needed to overcome all steps required for clinical application and drug development. As the research goes further deeply into DUBs molecular mechanisms, these questions will gradually be answered, and they will provide new ideas and new methods for the treatment of pathologies such as neurodegenerative and cancer diseases.

## Figures and Tables

**Table 1:** The seven classes of deubiquitinating enzymes (DUBs).

Species/DUBs	USP	OTU	JAMM	MJD	UCH	MINDY	ZUP1
*Homo sapiens*	56	17	12	4	4	5	1
*Danio rerio*	44	11	12	3	4	5	1
*Drosophila melanogaster*	23	5	10	1	3	1	0
*Schizosaccharomyces pombe*	11	1	4	0	2	1	1
*Saccharomyces cerevisiae*	11	3	7	0	1	1	0

## Data Availability

Not applicable.
